# Combination of Machine Learning and Analytical Correlations for Establishing Quantitative Compliance between the Trolox Equivalent Antioxidant Capacity Values Obtained via Electron Paramagnetic Resonance and Ultraviolet–Visible Spectroscopies

**DOI:** 10.3390/ijms231911743

**Published:** 2022-10-03

**Authors:** Eugene B. Postnikov, Mariola Bartoszek, Justyna Polak, Mirosław Chorążewski

**Affiliations:** 1 Theoretical Physics Department, Kursk State University, Radishcheva Str., 33, 305000 Kursk, Russia; 2 Institute of Chemistry, University of Silesia in Katowice, Ul. 9 Szkolna, 40-006 Katowice, Poland

**Keywords:** antioxidant capacity, wine, CatBoost machine learning, EPR spectroscopy, UV–vis spectrophotometry

## Abstract

Recent interest in the antioxidant capacity of foods and beverages is based on the established medical knowledge that antioxidants play an essential role in counteracting the damaging effects of free radicals, preventing human neurodegenerative diseases, cardiovascular disorders, and even cancer. At the same time, there is no “the method" that uniquely defines the antioxidant capacity of substances; moreover, the question of interrelation between results obtained by different experimental techniques is still open. In this work, we consider the trolox equivalent antioxidant capacity (TEAC) values obtained by electron paramagnetic resonance (EPR) spectroscopy and ultraviolet–visible (UV–vis) spectroscopy using the classic objects for such studies as an example: red, rosé, and white wine samples. Based on entirely different physical principles, these two methods give values that are not so simply interrelated; this creates a demand for machine learning as a suitable tool for revealing quantitative correspondence between them. The consideration consists of an approximate correlation-based analytical model for the key argument (i.e., $TEAC_{EPR}$) with subsequent adjustment by machine learning-based processing utilizing the CatBoost algorithm with the usage of auxiliary chemical data, such as the total phenolic content and color index, which cannot be accurately described by analytical expressions.

## 1. Introduction

A special medical interest [1,2,3,4] in antioxidants contained in food and beverages is substantiated by their essential role in counteracting the damaging effects of free radicals—which are known to cause aging and various diseases—by preventing their formation, scavenging them, or promoting their decomposition.

A suitable parameter that provides information on the effectiveness of antioxidants is the antioxidant capacity. Recently, there have been a variety of methods applied to evaluate antioxidant capacity, see e.g., the review in [5]. Nevertheless, it is worth mentioning that no simple universal method exists by which antioxidant activities can be measured accurately and quantitatively. One of the most popular methods for determining the trolox equivalent antioxidant capacity (TEAC) is the DPPH test [1]. DPPH (2,2-diphenyl-1-picrylhydrazyl) is a stable free radical whose alcoholic solution presents a deep purple color and a strong absorption band in the range of 515–520 nm. In the presence of antioxidant compounds, a DPPH radical can accept an electron or a hydrogen atom from the antioxidant scavenger molecule and is converted to a reduced form that is yellow. It is possible to determine antioxidant activity by studying the change of color spectrophotometrically. In comparison with some other tests based on single electron transfer [5], it has a number of advantages especially significant for testing foods and beverages at their normal consumable conditions: applicability at room temperature and normal pH conditions (in contrast, say, to ferric ion-based tests (FRAP) carried out in acidic pH conditions, and 2,2’-azinobis(3-ethylbenzthiazolin-6-sulfonic acid)-based test (ABTS), which is characterized by wide variation of typical times of reaction with different compounds, including phenolic ones, which leads to elevated uncertainty of results).

In addition, the chemical mechanism of substantiated DPPH is explored [6] from the point of view of reading the electron paramagnetic resonance (EPR) signals from molecules with unpaired electrons, i.e., from DPPH radicals and not from coexisting pigments. The latter may lead to the situation in which the conventional DPPH test based on measuring the absorbance with ultraviolet–visible spectroscopy (UV–vis) may be erroneous due to the overlap of DPPH absorption bands with the streaks of other components present in the substance studied [7,8]. Therefore, it seems that EPR is a more suitable technique for determining the total antioxidant capacity, but it is used much less frequently than the UV–vis method despite the fact that the value of total antioxidant capacity obtained by it is not affected by the turbidity or color of examined samples [7,8].

However, since both methods (UV–vis and EPR) address antioxidant efficiency towards the same compound, DPPH, one can naturally expect that the antioxidant capacity values determined via EPR and UV–vis spectroscopy should be correlated in general. On the other hand, it is a challenge to reveal whether these two independent techniques allow quantitative compliance of the TEAC values obtained.

At the same time, chemical and physical interactions and a huge number of factors involved make it practically impossible to build exact analytical models for the quantitative mapping between the numerical data that are outputs of these two different experimental methods of measurement. In turn, such problems that involve multiple ill-defined underlying elementary chemical and physical mechanisms can be attacked using machine learning, which operates with actually measurable data only.

In this work, we selected wine as the case study for application of such an approach because it represents a classic substance for which antioxidant capacity can vary over a very wide range resulting from various factors such as polyphenolic content, the color, the variety of grape, the method of wine production, sugar content, geographic region of origin of the grapes, and the method of aging. Thus, operating with such complex media can better highlight the specific issues related to the investigated problem. Further, antioxidant capacity of wines is of prominent medical and health science interest [9,10,11].

Recently, machine learning has taken a rapidly growing part in food and biochemical research due to the possibility of processing large datasets of parameters for which exact functional dependencies and their connection to target quantities are hard or even practically impossible to formulate explicitly [12,13]. Chemical analysis in wine studies is an exemplified case in this field. Machine learning supports analysis of wine authenticity, quality, geographical origin, and classification on the base of spectroscopic and chemical analytic properties of samples [14,15,16,17,18]. At the same time, a majority of these methods involve processing extensive datasets to simply predict a desired output in a “black box” manner. Simultaneously, there is modern understanding emerging [19] that machine learning can provide more enhanced opportunities in chemical characterization of compounds and interactions in the space of chemical parameters, with the subsequent development of relevant qualitative or analytical models. In particular, one can note some recent work related to wine studies, e.g., using Support Vector Machine (SVN)-based data processing to determine a non-linear model of the perception of wine astringency from its chemical composition [20], revealing best chemical parameters from a variety available to characterize a course of vine fermentation [21], and identifying the most important sensory descriptors discriminating the origin of wines [22]. Thus, it seems that machine learning is a prospective tool to find quantitative correspondence between $TEAC_{EPR}$ and $TEAC_{UV-vis}$. In addition, questions on how to account for other parameters characterizing antioxidant properties (TPC, CI, Tint) to achieve this goal should be posed.

## 2. Results

**Figure 1:**
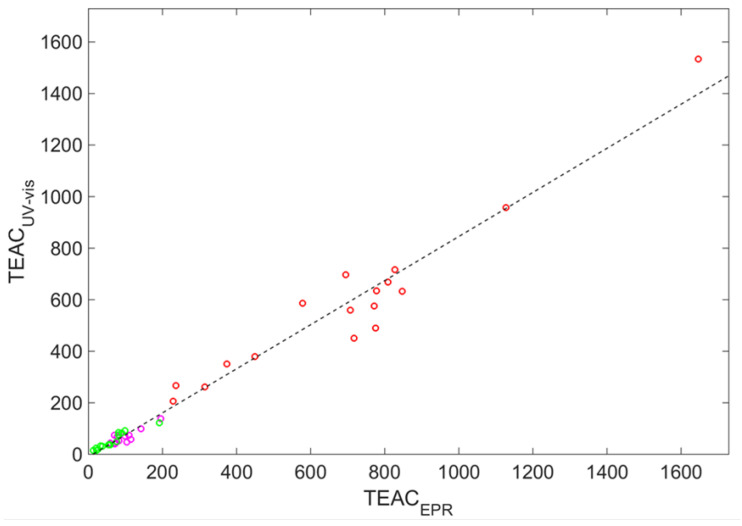
Experimental values of the trolox equivalent antioxidant capacity (TEAC) [μmolTE/100mL] obtained by UV and EPR measurements (circles), with the straight line representing a linear fit. Circle color indicates wine color: red (red wines), magenta (rose wines), and green (white wines).

Figure 1 demonstrates the interdependence between the raw $TEAC_{EPR}$ and $TEAC_{UV-vis}$ data. It is visible that there exists a correlation between them (the correlation coefficient $C_{corr}^{raw}=0.987$), but the markers are scattered around the straight fitting line (the correlation coefficient is equal to 0.987).

(1) $$TEAC_{UV-vis}^{fit}=0.8562TEAC_{EPR}-10.9811$$

**Figure 2:**
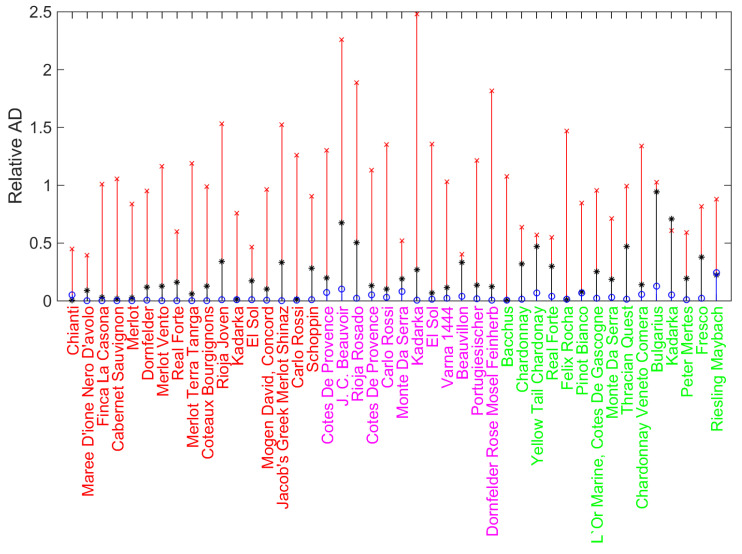
Relative absolute value deviations (AD) between predicted and experimental data on TEACUV–vis in the case of simple linear fit given by Equation (1) shown as stem plot with black asterisks as markers, and its enhancement with the CatBoost model supplied with the linear baseline (the stem plot with blue circles as markers). For comparison, the results of the “naïve” usage of machine learning, which does not take into the account the baseline correction, is shown as the stem plot with red crosses as markers. The color of the wines’ names indicates colors of wines: red (red wines), magenta (rose wines), and green (white wines).

The root-mean-square error (RMSE) is equal to 89.9μmolTrolox/100mL, and the maximal absolute deviation $AD_{max}=284.3\;\mu \mathrm{molTrolox}/100\;\mathrm{mL}$. The detailed distribution of the relative absolute deviations ($\left|TEAC_{UV-vis}^{predict}-TEAC_{UV-vis}\right|/TEAC_{UV-vis}$) respective to each variety of wine is shown in Figure 2 as the stem plot with asterisk markers.

This means that other parameters, which control these deviations from the fitting line, should be taken into consideration to improve the mapping between the two approaches for antioxidant capacity determination.

Thus, as the next step, we use the CatBoost regressing method, taking into account additional factors, both numerical (total phenolic content (TPC), color intensity (CI), tint (Tint) (i.e., the ratio of absorbances at 520 and 420 nm), and the content of alcohol) and categorical, i.e., defined as qualitative labels (sugar content (dry, semidry, semisweet, and sweet) and the geographical origin (at the level of countries)). The default limit of 1000 iterations is used to assure the convergence of the algorithm with respect to the RMSE. The procedure, following the workflow described in [23], consisted of two steps: (i) determining feature importance during fitting the model to the data and (ii) validation via subdivision of the given dataset into training and test sets with the complete permutation of all data.

Note that in contrast to standard cases of highly scattered data filling the parameter space, the presence of strong linear correlation (1) does not allow the simple direct training and prediction machine learning approach since it results in overfitting during the training and, subsequently, relatively large prediction error. The latter is significantly large for simple linear correlations; see the red-colored stem plot with crosses as markers in Figure 2.

To take the actual dependence between the key input parameter $TEAC_{EPR}$ and the output $TEAC_{UV-vis}$, we apply a more sophisticated method, which can be realized using CatBoost’s advantages: to use the so-called baseline feature, i.e., using data calculated via Equation (1) as an additional input dataset denoted as the baseline of the fitting procedure. Thus, in addition to elevating accuracy, such an approach also utilizes the approximate analytical dependence revealed independent of machine learning to correct the dependency, taking into account additional features for which analytical models are not explicitly defined.

As the first step, the self-consistency of the trained model and optimal parameters of tree-structured decomposition (the maximum depth of the trees) were determined using the whole sample as a training set. This procedure also determines the relative feature importance for each maximal tree depth as well as RMSE and maximal absolute deviations for each case. The results are listed in Table 1. One can see that the main parameters complementary to $TEAC_{EPR}$ are two color characteristics and the total phenolic content. The categorical features and the numerical feature of alcohol content play significantly lesser roles for all tree depths considered.

Thus, for the next test of CatBoost-based mapping between $TEAC_{EPR}$ and $TEAC_{UV-vis}$ data, we use only the four numerical parameters mentioned above and the baseline Equation (1). The testing procedure is organized as follows: we subsequently extract one representative wine from the dataset and use the remainder for model training; the trained model is applied to predict $TEAC_{UV-vis}$ using four input parameters for the wines not included in the training set. This method realizes the complete permutation of data and covers all wine samples, avoiding the drastic reduction of the training dataset’s size.

The results showing the prediction uncertainties are listed in Table 2 for different depths of trees. The best result is shown for a depth of 5, which is slightly deeper that the case of all parameters (see Table 1). This is explainable because categorical features have a small variety of values, while purely numerical consideration deals with more finely quantified numbers.

The resulting parameters characterizing the deviations between predicted and experimental data are RMSE=8.5μTrolox/100mL and $AD_{max}=50.8\;\mu \mathrm{Trolox}/100\;\mathrm{mL}$, i.e., the averaged prediction is ten times better than simple linear fitting, and the maximum deviation is more than five time less than without the machine learning-based adjustment. Figure 2 demonstrates this for each wine in the sample. One can see that the vast majority of circles are located significantly below the asterisks. This effect follows from the fact that CatBoost takes into account possible ranges of variables TPC, CI, and Tint for markers scattered around the fitting of the straight line in Figure 1. Figure 2 clearly demonstrates this for all examples of wines. The most notable is the drastic reduction of deviations for red wines (except Chianti); circles are uniformly placed practically on the line of zero deviations. This can originate from the fact that red wines have significant coloring and phenolic content, which provides valuable correction to the primary $TEAC_{UV-vis}$–$0.8562TEAC_{EPR}$ correlation. However, for rose and white wines, this effect is plausible too. The overall relative feature importances for the model build are: 34% ($TEAC_{EPR}$), 23.2% (TPC), 21.7% (Tint), and 21.1% (CI).

Note that TPC, Tint, and CI correlate with the main input parameter, $TEAC_{EPR}$, too. However, these correlations differ from the linear one expressed via Equation (1). They approximately have the power-law forms:

(2) $$TEAC_{EPR}=0.0432TPC^{1.32},$$

(3) $$TEAC_{EPR}=312.1Tint^{-1.44},$$

(4) $$TEAC_{EPR}=146.1CI^{0.65}.$$

**Figure 3:**
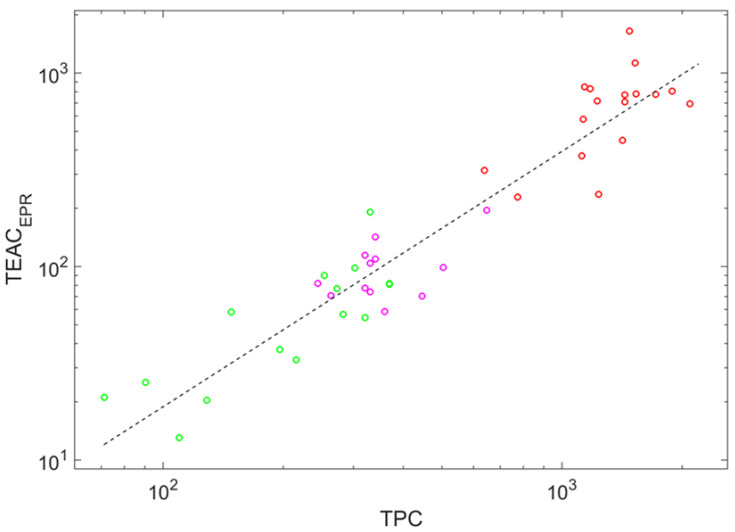
Double logarithmic plot showing the power-law dependence of the trolox equivalent antioxidant capacity determined by the EPR method on the total phenolic content; the dashed line shows the fit of these data stated by Equation (2). Circle color indicates wine color: red (red wines), magenta (rose wines), and green (white wines).

Among regularities stated as Equations (2)–(4), the most regular one corresponds to dependence on the total phenolic content; the correlation coefficient between $\ln(TEAC_{EPR})$ and ln(TPC) is 0.947 (see Figure 3, where markers form a relatively narrow stripe around the fitting line). This regularity supports the highest feature importance of this auxiliary parameter among three.

**Figure 4:**
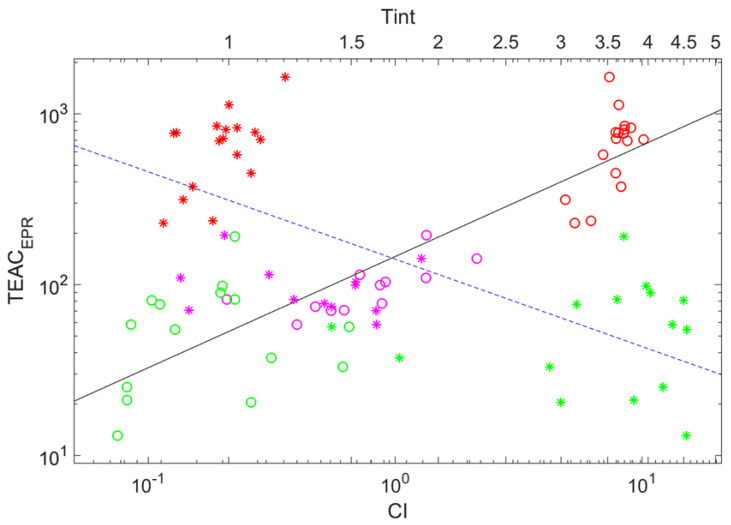
Double logarithmic plot showing the power-law dependencies of the trolox equivalent antioxidant capacity determined by EPR on the color intensity (circles, lower abscissa axis) and tint (asterisks, upper abscissa axis). The solid and the dashed lines denote fits stated by Equations (4) and (3), respectively. Circle color indicates wine color: red (red wines), magenta (rose wines), and green (white wines).

Figure 4 shows the same kind of plots for two auxiliary parameters characterizing wine’s coloring shown simultaneously. As is visible in the figure and from Equations (3) and (4), they behave alternatively to each other with the growth of the argument. There is some trend for both, but markers are scattered more that in the case of the dependence on TPC; the correlation coefficients between $\ln(TEAC_{EPR})$ and ln(Tint) and $\ln(TEAC_{EPR})$ and ln(CI) are equal to −0.696 (anti-correlation) and 0.893, respectively.

Thus, these dependencies allow better specifying of the target value $TEAC_{UV-vis}$. Note that the power-law functional form of Equations (2)–(4) differs from the linear trend of the principal argument $TEAC_{EPR}$; Equation (1), and, moreover, the power indices in Equations (2)–(4) are also different. This fact assures that all four input variables do not form a linearly-dependent system, and each one makes its own input into the prediction by subsequent adjustment of the parametric space by the cascade of multiple decision trees that is the essence of the CatBoost algorithm.

## 3. Discussion

All the wines studied exhibited antioxidant properties. The values of $TEAC_{EPR}$ were within the range 13.04–1646.71 μmolTrolox/100mL, which is consistent with other reports found in the literature [24,25,26]. Such a wide range of $TEAC_{EPR}$ values indicates a very diverse group in terms of their antioxidant properties. It should be noted that red wines are characterized by an extensive range of TEAC (from 236.70 to 1646.71 μmolTrolox/100mL). The predominant antioxidant species in red wines are phenolic compounds [27], mainly flavonoid (anthocyanins, flavonols, and flavanols, proanthocyanidins) and non-flavonoid compounds (phenolic acids) [28]. Moreover, it is worth noting that anthocyanins are the main composition with significant contributions to the antioxidant capacity of red wine [29]. This fact may explain the considerable discrepancy between $TEAC_{EPR}$ and $TEAC_{UV-vis}$ values as anthocyanins exhibit the absorbance maximum at around 520 nm ($\lambda_{max}$ for DPPH is 517 nm). In the case of white wine, the molecular fraction associated with oxidative stability is not well established. However, recent studies have shown that nitrogen- and sulfur-containing compounds are the main contributors to the antioxidant metabolome of white wine [27]. These compounds do not significantly affect the absorbance value measured during the determination of TEAC spectrophotometrically.

Thus, we need to highlight the fact that such a correlation may lead to poor results, when the “naïve” usage of machine learning, which does not take dependencies between input parameters, may lead not only to poor understandable but even to wrong results. On the contrary, the two-step procedure, which reveals, as the first step, possible analytical correlations between control factors that are not accurate enough to give the acceptable quantitative mapping between the data obtained by two methods influenced by different physical and chemical underlying mechanisms but state some trends, supplied with the second step of refining the desired mapping, leads to the results establishing the target interdependence quantitatively.

Recently, the role of products showing antioxidant properties has been the subject of growing interest in dietetics, protective therapy, and treatment of many diseases. In large part, these compounds are not synthesized by the human physiological system, so their delivery with food is essential, especially for protection against free radicals. Consequently, investigations focused on nutrition and drinks containing antioxidants, such as vitamins, flavonoids, catechins, and other natural antioxidants, which evidently can prevent diseases, are in high demand. However, the values of the antioxidant capacity obtained in different laboratories can be different even for the same products due to different methods of analysis and biomarkers on which they are focused. Thus, one need methods able to establish compliance between such a variety of data. As demonstrated in our work, the special combination of analytical and machine learning-based approaches opens the way for standardization of antioxidant characteristics for the food and beverage industry within the context of healthy diet standards.

## 4. Materials and Methods

### 4.1. Samples, Chemicals, and Experimental Data

A total of 44 wines samples, 17 red, 12 rosé, and 15 white, were purchased from local markets. The detailed list of samples, including sample information, type of wine, content of alcohol, origin, and year of production, is shown in the Supplementary Information (Table S1). Samples were opened, protected against sunlight, and stored at $4{\;}^{\circ }C$. 1,1-diphenyl-2-picrylhydrazyl (DPPH•) (Sigma-Aldrich, Poznań, Poland) was used as the source of free radicals. To quantify the antioxidant activity of wine, trolox (molecular formula C14H18O4) (Acros Organics, Geel, Belgium) was used. In order to determine total polyphenol content, FC reagent and gallic acid (GA) (P.O.Ch., Gliwice, Poland) were used. All other chemicals and solvents were of analytical grade and were used without further purification.

Antioxidant capacity was determined using the method described previously [30]. Electron paramagnetic resonance spectra were obtained with a Bruker EMX EPR spectrometer (Bruker-Biospin, Germany) operating at the X-band frequency at room temperature, and $TEAC_{UV-vis}$ was determined using the DPPH method performed at 515 nm using a Lambda Bio 40 spectrophotometer (Perkin Elmer, USA). Measurement of additional factors used for machine learning included total phenolic content (TPC) determined with the Folin–Ciocalteu method using gallic acid as the standard [31]. Color intensity (CI) was measured using a Lambda Bio 40 spectrophotometer and calculated as the sum of absorbances at 420, 520, and 620 nm. Tint was measured using a Lambda Bio 40 spectrophotometer and calculated as the ratio of absorbances at 520 and 420 nm. The details of experimental procedures are provided in the Supplementary Information.

### 4.2. Machine Learning Method and Data Analysis

Among various existing approaches, we chose the CatBoost algorithm based on gradient boosting on decision trees; it was developed by the Yandex Corporation relatively recently [32] and made publicly available as open-source software (https://catboost.ai/, accessed on 28 September 2022). This choice was motivated by its prospective features such as native support of categorical and heterogeneous (mixed numerical and categorical) data using originally ordered target statistics. This has led to growing popularity of this machine learning solution for classification and regression in a wide variety of interdisciplinary applications; for review of the recent state-of-the-art, see [33]. Another useful feature following from this novel implementation of ordered statistics/boosting is the direct possibility to explicitly ascertain the relative importance of input parameters, which replaces “black box” machine learning by analysis of, say, chemical or biochemical premises of the principal control quantities for subsequent model building [23,34].

We operated with the standalone command-line binary version 0.26 of CatBoost for Windows. The formation of csv-formatted tables of input quantities and further analysis and plotting of the output csv-formatted data were carried out using MATLAB (version R2014b was used, but the code contains only standard core functions, which are version-independent). The complete set of files, named according to the numeration of figures in this work, can be accessed at https://github.com/postnicov/TEACdataprocessing, accessed on 28 September 2022.

## 5. Conclusions

The results presented in this study highlight that the CatBoost algorithm is a novel, forward-looking tool suitable for chemistry applications. Its native support of the combination of numerical and categorical features and its baseline correction make it attractive for solving two tasks: (i) when the system is characterized by not only quantitative (numerical) but also qualitative (categorical) parameters and one needs to estimate relative importance of both types of inputs and (ii) when there is a strong correlation between numerical parameters that should be taken into account for formulating the task for machine learning.

Here, we demonstrated this specificity by addressing the quantitative compliance of the TEAC values obtained using two independent techniques with the auxiliary usage of data of chemical and spectroscopic analysis of different wines. The wine studies allowed highlighting of both of the principal features mentioned above. First of all, these fluids are highly characterized by categorical features such as the place of origin, sugar content determined ubiquitously by the qualitative scale, etc. Thus, the problem was whether such data could be used during the quantitative predictive procedure without additional detailed chemical analysis and the range of their importance. Note that wine is not a unique example in this sense; another typical categorical feature widespread in chemical studies is, e.g., the set of chemical groups. Another important conclusion draws attention to the presence of correlations between data in the regression problems. Neglecting such correlations can lead to extremely high uncertainty in the predictions, but improvement when a trend is explicitly included in consideration, and leaves refining the prediction of unordered scattered data to the machine learning part of study, i.e., the task which it is most suitable for.

As a result, the root mean squared deviation between the actual and predicted data is ten times diminished in comparison to the simple correlation and almost five times respective to the maximal absolute deviation; the improvement is even better comparing to the naïve ML-prediction, which does not take into account the correlations between the data.

Finally, the results of this work are not limited by these methodological demonstrations; we also have reported the datasets of the trolox equivalent antioxidant capacity obtained by the conventional DPPH test as well as by the more modern EPR-based approach and demonstrated how they can mapped to each other.

## Figures and Tables

**Table 1:** Uncertainties of the model’s fitting and feature importance (%) and for different depths of trees used for decomposition.

Tree Depth	2	3	4	5
RMSE, μTrolox/(100mL)	18.9	17.6	18.1	21.2
Max(AD), μTrolox/(100mL)	56.3	68.1	74.8	90.3
$TEAC_{EPR}$	40.0	40.2	35.4	32.5
Tint	16.0	14.9	15.1	15.1
TPC	15.8	15.5	13.3	14.1
CI	15.6	12.8	16.7	13.3
Origin	5.7	7.2	7.9	11.3
AlcContent	4.6	5.1	6.9	6.4
Sugar	2.4	4.2	4.7	7.3

**Table 2:** Prediction uncertainties with different tree decomposition depths for validating the model with four numeral input parameters and the linear baseline equation.

Tree Depth	RMSE, μmolTrolox/(100mL)	Max(AD), μmolTrolox/(100mL)
3	13.5	75.8
4	11.4	60.3
5	8.5	50.8
6	11.9	66.3

## Data Availability

The detailed description of the experimental methods and the full set of data obtained are provided online in the supporting information file https://www.mdpi.com/article/10.3390/ijms231911743/s1; the complete set of program codes used for calculations named according to the numeration of figures in this work can be accessed at https://github.com/postnicov/TEACdataprocessing, accessed on 28 September 2022.

## Supplementary Materials

The following are available online at https://www.mdpi.com/article/10.3390/ijms231911743/s1.
