# Supplementary material for: Combination of Machine Learning and Analytical Correlations for Establishing Quantitative Compliance between the Trolox Equivalent Antioxidant Capacity Values Obtained via Electron Paramagnetic Resonance and Ultraviolet–Visible Spectroscopies
Source: Int J Mol Sci. 2022 Oct 3;23(19):11743. doi: 10.3390/ijms231911743 (PMC9569636; doi:10.3390/ijms231911743)
Supplement: Supplementary file 1 [file ijms-23-11743-s001.zip › ijms-1952063-supplementary.pdf]

Supplementary Information

Combination of machine learning and analytical correlations for establishing quantitative compliance between the the Trolox equivalent antioxidant capacity values obtained with two independent techniques

Eugene B. Postnikov*, Mariola Bartoszek**, Justyna Polak**,

Mirosiaw Chor№ïewski***

*Department of Theoretical Physics, Kursk State University, Radishcheva 33, 305000 Kursk, Russia; postnikov@kursksu.ru

**Faculty of Science and Technology, Institute of Chemistry, University of Silesia, Szkolna 9, 40-006 Katowice, Poland

***Faculty of Science and Technology, Institute of Chemistry, University of Silesia, Szkolna 9, 40-006 Katowice, Poland; miroslaw.chorazewski@us.edu.pl

Determination of antioxidant capacity by the EPR spectroscopy

Antioxidant capacity was determined using the method described previously. (15) Electron paramagnetic resonance spectra were obtained with a Bruker EMX EPR spectrometer (Bruker-Biospin, Germany) operating at the X-band frequency at room temperature. The typical instrument parameters were: central field, 3480 G; modulation amplitude, 2.0 G; time constant, 40.96; gain, $1*10^4$ G; microwave power, 20.12 mW. The regression equation for the linear relationship between the percent inhibition of EPR signal intensity and the mol number of trolox was assessed as: y=1547.8x+4.7, where: y is the inhibition [%] and x is the volume of the sample [mL]. This equation was used to calculate the antioxidant activity of the studied samples in μmol trolox per 100 mL of the studied samples.

A typical reaction mixture contained 1 mL of 200 μmol/L $DPPH^•$ solution in ethanol together with 0.0003 mL to 0.380 mL of sample, depending on the manifested antioxidant properties. For all samples, regression equation of the linear relationship between the percent inhibition (*%I*) of the EPR signal intensity and the volume of sample (*V*) was determined. Based on this equation the *%I* corresponding to 100 mL of the studied sample was calculated. Then, from the standard curve obtained for trolox, the antioxidant activity given in μmol trolox per 100 mL of sample was defined. The data presented here are the result of three trials.

Determination of antioxidant capacity by UV-vis spectroscopy

$TEAC_{UV\text{-}vis}$ was determined using the DPPH method. (15) UV-vis spectrophotometric measurements were performed at 515 nm using Lambda Bio 40 spectrophotometer (Perkin Elmer,USA). On

increasing the concentration of trolox, the intensity of the corresponding value of absorbance decreased and consequently, the percent inhibition (*%I*) increased. The regression equation of the linear relationship of the percent inhibition of absorbance to mol number of trolox was used to calculate the antioxidation activity of the studied samples in μmol trolox /100 mL of the studied sample. The regression equation for the linear relationship between the percent inhibition of absorbance and the mol number of trolox was assessed as: $y = 1491x + 0.5$, where: y is the inhibition [%] and x is the volume of the sample [mL]. This equation was used to calculate the antioxidant activity in μmol trolox per 100 mL of the studied samples (μmol TE/100 mL).

The percent inhibition of the decrease in absorption at 515 nm calculated according to the following equation: $\% \ Inhibition = [(A_0 - A)/A_0]*100\%$, where A_0 is the absorbance of DPPH$^\bullet$ (control sample), and A is the absorbance of DPPH$^\bullet$ with a sample.

A typical reaction mixture contained 3 mL of the 200 μmol/L DPPH$^\bullet$ solution in ethanol together with 0.0009 mL to 1.140 mL of wine. For all samples regression equation of linear relationship of the percent inhibition (*%I*) of the absorbance to the volume of sample (*V*) was determined. On the basis of this equation *% I* corresponding to 100 mL of the studied sample was calculated. Then from the standard curve the antioxidant activity μmol Trolox per 100 mL of sample (μmol TE/100 mL) was defined. The presented data are the means of three determinations.

Determination of Total Phenolic Content

The total phenolic content (TPC) was determined with Folin-Ciocalteu method using gallic acid as the standard. This method measures the total available hydroxyl groups. At the reaction pH, phenols in the sample are present in the form of the phenolate ion, which can reduce the Folin–Ciocalteu reagent. The absorbance was monitored at 765 nm using Lambda Bio 40 spectrophotometer. The concentration of the total phenolics was calculated as mg of gallic acid equivalent by using an equation obtained from gallic acid calibration curve and the results were expressed as mg gallic acid per litre of wine (mg GAE/L). The data presented here are the result of three trials.

Determination of Color Intensity and Tint

Color intensity (*CI*) was measured using Lambda Bio 40 spectrophotometer and calculated as the sum of absorbances at 420, 520 and 620 nm.

Tint was measured using Lambda Bio 40 spectrophotometer and calculated as the ratio of absorbances at 520 and 420 nm.

Table S1. An overview of the wine samples investigated. Antioxidant capacity measured by EPR and UV-vis spectroscopy, total phenolic content and color intensity of tested wines.

No	Sample	Origin	Color	Wine type	Year	Content of alcohol [%]	$TEAC_{EPR}$ [мmol TE/100 mL]	$TEAC_{UV-vis}$ [мmol TE/100 mL]	TPC [mgGA/L]	CI	Tint
1	Chianti	Italy	red	dry	2014	12.5	1127.04	958.14	1523.08	8.05	1.03
2	Maree D'ione Nero D'avolo	Italy	red	dry	2013	13	1646.71	1534.40	2575	7.36	0.89
3	Finca La Casona	Spain	red	dry	2014	12	778.03	634.72	1532.69	7.82	0.96
4	Cabernet Sauvignon	France	red	dry	2014	12.5	808.32	667.56	1888.46	8.49	0.97
5	Merlot	France	red	dry	2013	12.5	827.33	716.08	1176.92	9.03	1.11
6	Dornfelder	Germany	red	dry	2012	12.5	373.56	350.97	1119.23	8.23	0.83
7	Merlot Vento	Italy	red	dry	2014	12	847.51	632.93	1138.46	8.51	0.98
8	Real Forte	Portugal	red	dry	2014	13.5	694.16	696.54	2090.38	8.72	0.86
9	Merlot Terra Tanrga	Bulgaria	red	dry	2013	13	707.51	560.09	1436.54	10.16	1.03
10	Coteaux Bourgignons	France	red	dry	2014	12.5	771.35	575.91	1436.54	8.42	0.81
11	Rioja Joven	Spain	red	dry	2014	13.5	716.50	450.10	1225	7.84	0.84
12	Kadarka	Bulgaria	red	semisweet	2015	11.5	314.58	262.19	638.46	4.90	1.08
13	El Sol	Spain	red	semisweet	2014	10.5	577.62	585.83	1128.85	6.96	0.95

Abbreviations: $TEAC_{EPR}$ - Trolox equivalent antioxidant capacity determined by EPR spectroscopy, $TEAC_{UV-vis}$ - Trolox equivalent antioxidant capacity determined by UV-vis spectroscopy, TPC – Total Phenolic Content, CI – color intensity of wine ($CI = A_{420} + A_{520} + A_{620}$ (where $A_{л}$ represents the absorbance at wavelength л)

14	Mogen David, Concord	USA	red	semisweet	2014	11	228.73	206.96	773.08	5.34	1.40
15	Jacob's Greek Merlot Shinaz	Australia	red	semisweet	2014	11.7	774.68	490.32	1715.38	8.01	1.52
16	Carlo Rossi	USA	red	sweet	2013	9.5	448.73	379.86	1417.31	7.81	1.14
17	Schoppin	Bulgaria	red	sweet	2015	12	236.70	267.48	1234.62	6.20	1.24
18	Cotes De Provence	France	rose	dry	2014	12.5	74.17	44.36	330.77	0.47	1.63
19	J. C. Beauvoir	France	rose	dry	2012	12.5	103.89	46.89	330.77	0.91	1.37
20	Rioja Rosado	Spain	rose	dry	2013	12.5	114.37	58.18	321.15	0.72	0.88
21	Cotes De Provence	France	rose	dry	2014	12.5	81.98	52.83	244.23	0.21	1.52
22	Carlo Rossi	USA	rose	semidry	2014	9.5	58.43	44.28	359.62	0.40	1.89
23	Monte Da Serra	Portugal	rose	semidry	2013	12.5	77.37	69.23	321.15	0.88	1.63
24	Kadarka	Bulgaria	rose	semisweet	2015	11	70.96	39.77	263.46	0.62	0.99
25	El Sol	USA	rose	semisweet	2014	10.5	99.13	69.74	503.85	0.87	0.85
26	Varna 1444	Bulgaria	rose	semisweet	2012	11	141.88	99.75	340.38	2.14	1.41
27	Beauvillon	France	rose	semisweet	2014	11.5	70.18	74.52	446.15	0.55	1.76
28	Portugiesischer	Portugal	rose	semisweet	2014	9.5	195.34	137.90	648.08	1.34	2.89
29	Dornfelder Rose Mosel Feinherb	Germany	rose	semisweet	2015	10.5	109.24	74.08	340.38	1.33	3.61
30	Bacchus	Poland	white	dry	2013	10	56.45	37.84	282.69	0.65	4.55

31	Chardonnay	Poland	white	dry	2014	12	37.22	31.67	196.15	0.32	4.33
32	Yellow Tail Chardonay	Australia	white	dry	2010	13	32.93	33.90	215.38	0.61	3.69
33	Real Forte	Portugal	white	dry	2014	13	81.80	84.92	369.23	0.22	3.97
34	Felix Rocha	Portugal	white	dry	2014	12	54.47	36.75	321.15	0.13	3.81
35	Pinot Bianco	Italy	white	dry	2014	12	58.30	36.77	148.08	0.09	3.16
36	L`Or Marine, Cotes De Gascogne	France	white	dry	2014	11.5	191.56	122.70	330.77	0.22	4.54
37	Monte Da Serra	Portugal	white	semidry	2015	12.5	98.42	91.05	301.92	0.20	3.00
38	Thracian Quest	Bulgaria	white	semidry	2015	10.5	21.10	14.68	71.15	0.08	4.03
39	Chardonnay Veneto Comera	Italy	white	semidry	2015	12	76.75	48.56	273.08	0.11	4.20
40	Bulgarius	Bulgaria	white	semisweet	2015	11	13.04	15.35	109.62	0.08	4.50
41	Kadarka	Bulgaria	white	semisweet	2015	11	20.41	24.85	128.85	0.26	1.03
42	Peter Mertes	Germany	white	semisweet	2014	9.5	89.96	82.64	253.85	0.20	0.89
43	Fresco	Poland	white	semisweet	2015	10	25.14	18.04	90.38	0.08	0.96
44	Riesling Maybach	Grmany	white	semisweet	2014	10	80.92	75.92	369.23	0.10	0.97
